# Structure–Property–Function Relationships in Stimuli-Responsive Hydrogels for Brain Organoid Vascularization

**DOI:** 10.3390/gels12040287

**Published:** 2026-03-29

**Authors:** Minju Kim, Hoon Choi, Woo Sub Yang, Hyun Jung Koh

**Affiliations:** 1 Department of Anesthesiology and Pain Medicine, Seoul St. Mary’s Hospital, College of Medicine, The Catholic University of Korea, 222, Banpo-daero, Seocho-gu, Seoul 06591, Republic of Korea; jkkpsk@gmail.com (M.K.); hoonie83@catholic.ac.kr (H.C.); 2 Department of Genetics, Yale Stem Cell Center, Yale School of Medicine, New Haven, CT 06520, USA; woosubyang@gmail.com

**Keywords:** brain organoids, dynamic biomaterials, neurovascular unit, spatiotemporal control, stimuli-responsive hydrogels, structure–property–function framework, vascularization

## Abstract

Human induced pluripotent stem cell (iPSC)-derived brain organoids have emerged as powerful three-dimensional (3D) platforms for modeling human neurodevelopment and neurological disorders. However, the absence of a functional vascular network remains a critical limitation, restricting oxygen and nutrient delivery, impairing metabolic stability, and constraining long-term maturation. Conventional extracellular matrix (ECM) mimetics, such as Matrigel and other static synthetic hydrogels, provide biochemical support but fail to recapitulate the dynamic remodeling that characterizes the developing neurovascular niche. Recent advances in stimuli-responsive hydrogels offer spatiotemporal control over matrix stiffness, degradability, viscoelasticity, and biochemical cue presentation. In this review, we discuss dynamic hydrogel systems within a structure–property–function framework, highlighting how network chemistry and architecture may regulate endothelial sprouting, lumen formation, vascular stabilization, and neurovascular unit maturation in vascularized brain organoid models, based on evidence from both organoid studies and related biomaterial or vascular systems. Photoresponsive, enzyme-cleavable, thermo-responsive, supramolecular, bio-orthogonal click-based, and bioprinted platforms are discussed with emphasis on mechanotransduction, angiocrine signaling, and barrier specialization. Functional outcomes, including trans-endothelial electrical resistance, selective permeability, transporter expression, electrophysiological integration, and sustained perfusion, are discussed alongside translational challenges such as cytocompatibility, oxidative stress, scalability, and regulatory feasibility. Collectively, dynamic hydrogels provide a versatile biomaterial strategy for improving vascularization and aspects of functional maturation in brain organoid models with enhanced physiological relevance. Ultimately, stimuli-responsive hydrogel systems may serve as enabling platforms for engineering vascularized brain organoids and advancing human-relevant neurovascular disease modeling.

## 1. Introduction

### 1.1. The Promise and Challenges of Brain Organoids

Human induced pluripotent stem cell (iPSC)-derived brain organoids have emerged as a revolutionary 3D in vitro platform, providing unprecedented opportunities to recapitulate human neurodevelopment and model complex neurological diseases [1]. Unlike traditional 2D cultures, these organoids mimic the spatial organization and cellular heterogeneity of the developing brain [2,3]. However, a critical bottleneck remains: the lack of a functional vascular network [1,4]. In vivo, the brain is a highly metabolic organ where oxygen and nutrients are supplied through an intricate vascular system [4]. In contrast, current brain organoids rely solely on passive diffusion, which becomes insufficient beyond ~350 μm from the organoid surface, often resulting in central necrosis within the organoid core [5], leading to the formation of a necrotic core at the center and limiting the overall physiological relevance and maturational potential of the tissues [6,7]. Unlike other organoid systems, brain organoids must support neural progenitor maintenance, neuronal migration, astrocyte maturation, and long-term electrophysiological activity. These requirements impose additional constraints on hydrogel design beyond merely matching the extreme softness of brain tissue.

### 1.2. The Limitations of Static Extracellular Matrix (ECM) Mimics

Conventionally, brain organoids have most commonly been cultured in relatively static natural extracellular matrix mimics, most notably Matrigel [8]. While these matrices provide essential biochemical cues, they incompletely replicate the spatiotemporal and cell-instructive complexity of the native brain ECM. Static scaffolds possess fixed mechanical properties and uncontrollable degradation rates, which often impede the dynamic processes of angiogenesis and neurogenesis [9,10]. The matrix stiffness that is initially permissive for early cell seeding may later become restrictive, limiting organoid expansion, endothelial cell infiltration, and the dynamic cell-matrix interactions required for neurovascular development as the tissue matures [9,11].

Nevertheless, static ECM mimics provide several important advantages for organoid culture. Natural matrices such as Matrigel contain a complex mixture of extracellular matrix proteins and growth factors that support cell survival, adhesion, and early neural progenitor expansion. In addition, these systems are widely adopted due to their ease of use, experimental reproducibility, and compatibility with established brain organoid culture protocols [10,12].

### 1.3. The Paradigm Shift: Toward Dynamic Biomaterial Platforms

To overcome these spatiotemporal limitations, a paradigm shift from static to dynamic biomaterials is essential [13]. The native ECM is not a static scaffold but a highly responsive environment that undergoes constant enzymatic remodeling and biochemical signaling [14]. In this context, stimuli-responsive or “smart” hydrogel systems—whose properties can be tuned on-demand via external triggers such as light, temperature, or enzymatic activity—offer a promising solution [15,16]. Commonly used biomaterials for constructing such dynamic hydrogels include synthetic polymers such as polyethylene glycol (PEG), as well as naturally derived matrices, including hyaluronic acid, gelatin methacrylate (GelMA), and alginate, which provide tunable biochemical and mechanical properties suitable for organoid culture systems [17,18]. By modulating hydrogel stiffness and degradation kinetics in a spatiotemporal manner, these systems may help guide early endothelial infiltration and subsequently support vascular stabilization during later maturation stages [14,19].

### 1.4. Scope of This Review

In this review, we examine brain organoid vascularization through a structure–property–function framework, focusing on how hydrogel chemistry and network design shape matrix mechanics, transport behavior, and remodeling, which in turn influence endothelial and neurovascular outcomes [14]. We further discuss how spatiotemporal control of biomaterial properties may facilitate the neurovascular unit (NVU) and improve the physiological relevance of 3D brain models [20]. Finally, we discuss the current challenges and future perspectives of integrating dynamic platforms into organ-on-a-chip technologies to achieve a new level of fidelity in human brain modeling [14,21].

## 2. Limitations of the Static ECM Paradigm

The “static scaffold paradigm” refers to ECM mimetics (e.g., Matrigel, simple PEG hydrogels) whose biochemical presentation, mechanical properties, and degradation behavior remain largely fixed throughout culture [22,23]. Such invariability limits the adaptive remodeling and mechanical feedback required for coordinated vascular integration [11,24].

### 2.1. Matrigel-Based Systems: Variability and Structural Passivity

Although Matrigel is widely used in brain organoid culture, it poses inherent limitations for the generation of vascularized constructs [25]. Batch-to-batch variability in protein composition (laminin, collagen IV, etc.) reduces experimental reproducibility [26,27]. Furthermore, limited dynamic remodeling restricts matrix reorganization in response to evolving tissue demands, which may compromise long-term structural adaptability, endothelial integration, and neural maturation [11,22,23].

Nevertheless, Matrigel remains advantageous for early-stage organoid formation because it contains a complex mixture of ECM proteins and growth factors that support cell survival, adhesion, and initial tissue patterning.

### 2.2. Simple Hydrogel Encapsulation: Diffusion and Stiffness Mismatch

Conventional encapsulation strategies depend primarily on passive diffusion for oxygen and nutrient transport, creating a well-recognized bottleneck in larger organoid constructs [28]. In organoids exceeding 150–200 μm in thickness, diffusion limitations can produce severe hypoxia and eventual necrotic core formation [29,30]. Furthermore, a substantial stiffness mismatch often exists; many static hydrogels do not reproduce either the extreme compliance of the developing brain or the dynamic mechanical evolution associated with neurovascular maturation, which may limit physiologically relevant endothelial invasion and tissue organization [23,31].

**Figure 1:**
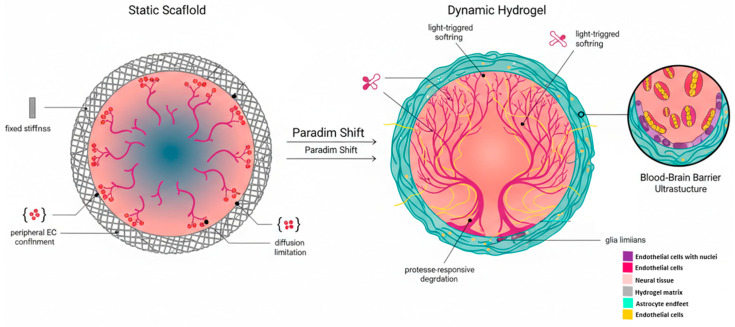
The paradigm shift from static to dynamic environments. Schematic representation of the transition in brain organoid engineering. (**Left**) Static scaffold with fixed mechanical properties imposes diffusion constraints and restricts endothelial infiltration, contributing to central hypoxia and necrosis. (**Right**) Dynamic, stimuli-responsive hydrogels facilitate synchronized vascularization and neural growth through on-demand stiffness modulation and biochemical adaptability.

Collectively, these limitations underscore the need for a paradigm shift from structurally passive matrices toward dynamically instructive biomaterial platforms. This conceptual transition is schematically illustrated in Figure 1.

Building upon this framework, the following section discusses stimuli-responsive mechanisms that enable such dynamic remodeling.

Table 1 summarizes the fundamental differences between conventional static matrices and emerging dynamic platforms, emphasizing the transition from passive scaffolds to instructive environments that actively guide neurovascular maturation.

This comparison highlights key aspects such as mechanical properties, matrix remodeling, vascular sprouting, nutrient and oxygen supply, biochemical signaling, biological relevance, and major challenges.

Despite these limitations, simple hydrogel encapsulation strategies provide several practical advantages, including tunable polymer chemistry, reproducible fabrication, and compatibility with defined synthetic matrices that reduce batch variability.

## 3. Stimuli-Responsive Mechanisms and Engineering of Dynamic Hydrogels

Vascularization is generally understood as a spatiotemporal process influenced by matrix softening, degradation, and growth factor gradients. In this context, adaptive hydrogel matrices serve as instructive environments that help guide this process through externally or internally triggered changes in matrix properties.

Examples of polymers capable of undergoing structural modification in response to external stimuli include PEG-based hydrogels incorporating photocleavable groups, poly (N-isopropylacrylamide) (PNIPAM) thermo-responsive polymers, and host–guest supramolecular networks based on cyclodextrin–adamantane interactions. These polymers provide a versatile platform for engineering dynamic microenvironments capable of guiding endothelial morphogenesis and neurovascular unit formation.

### 3.1. Photoresponsive Mechanisms: Spatiotemporal Patterning

Photoresponsive hydrogel platforms, such as those incorporating photocleavable o-nitrobenzyl (oNB) moieties or photopolymerizable methacrylated hyaluronic acid (HAMA), enable precise spatiotemporal modulation of the matrix environment. By utilizing light-induced cleavage (e.g., via UV light) or radical polymerization (e.g., via visible-light and lithium phenyl-2,4,6-trimethylbenzoylphosphinate (LAP) initiators), these systems allow for in situ tuning of crosslink density and elastic modulus without compromising global matrix integrity [17,32,33,34].

Such photochemical control can be used to generate defined microchannels and local stiffness gradients that are expected to support endothelial invasion and vascular stabilization. However, much of this mechanistic rationale has been established in related biomaterial or vascular models, and direct validation in vascularized brain organoids remains comparatively limited [35,36]. This on-demand adaptability may help align hydrogel architecture with the evolving metabolic and structural demands of the developing organoid systems.

### 3.2. Enzyme-Triggered Remodeling: Cell-Instructed Bio-Responsiveness

Enzyme-responsive hydrogel networks incorporate matrix metalloproteinase (MMP)-cleavable peptide sequences (e.g., GPQGIWGQ) as degradable crosslinkers within PEG- or gelatin-based networks. These linkers are selectively hydrolyzed by endothelial cell (EC)-secreted MMP-2 and MMP-9, resulting in localized crosslink scission rather than bulk matrix degradation [37,38]. By coupling protease sensitivity to network architecture, these systems link peptide sequence design and crosslink density to local matrix softening, permeability, and remodeling kinetics. Because protease activity is spatially confined to invading ECs, matrix remodeling can become cell-instructed and self-regulated, thereby recapitulating key features of in vivo angiogenic invasion in related 3D vascular models [37,39]. Importantly, the degradation kinetics can be modulated by peptide sequence design and crosslink density, enabling quantitative control over sprouting speed, lumen formation, and network stability. For example, commonly used MMP-sensitive peptide sequences such as GPQGIWGQ contain a specific cleavage site recognized by MMP-2 and MMP-9, resulting in relatively rapid hydrogel degradation. In contrast, alternative sequences such as VPMSMRGG exhibit slower enzymatic cleavage rates, enabling more gradual matrix remodeling. By selecting different peptide cleavage motifs, the degradation kinetics of PEG-based hydrogels can therefore be tuned to better match endothelial invasion dynamics and angiogenic sprouting behavior in three-dimensional culture systems. Such protease-dependent remodeling is conceptually attractive for vascularized brain organoids, where synchronized ECM softening and endothelial invasion are expected to support coordinated NVU assembly, although direct organoid-specific evidence remains limited [38,40].

### 3.3. Thermo-Responsive and Supramolecular Networks: Physical Reversibility

Physical reversibility introduces dynamic stress dissipation and network rearrangement, enabling matrices to accommodate both initial cell encapsulation and subsequent cell migration.

#### 3.3.1. Thermo-Responsive Systems

By leveraging a sol–gel transition near physiological temperature (37 °C), these systems ensure a homogeneous 3D distribution of cells with minimal damage [41]. Beyond encapsulation, temperature-driven network rearrangements may modulate pore structure and diffusivity, indirectly influencing angiogenic initiation through altered oxygen and nutrient transport [42,43].

#### 3.3.2. Supramolecular (Reversible) Chemistry

Supramolecular hydrogel systems are constructed through dynamic non-covalent interactions, including host–guest inclusion complexes, ionic interactions, and hydrogen bonding. In β-cyclodextrin-based systems, hydrophobic adamantane groups reversibly bind within the cyclodextrin cavity (association constants ~10^4^–10^5^ M^−1^), enabling rapid bond dissociation and reformation under mechanical stress. These reversible interactions confer shear-thinning and self-healing behavior [44]. Importantly, such networks can dissipate forces generated by migrating endothelial cells during sprouting, absorbing stress, and reforming crosslinks rather than behaving as rigid mechanical barriers [36]. Because crosslinks are physically defined rather than covalent, the matrix can undergo fluid-like rearrangement while preserving overall structural integrity [45,46]. This tunable stress relaxation enables dynamic cell-instructed remodeling relevant to vascular morphogenesis in three-dimensional culture systems.

### 3.4. Bio-Orthogonal Chemistry and 3D Bioprinting Strategies

Beyond light and enzymes, bio-orthogonal or click chemistry provides a highly selective means to modulate hydrogel properties in situ. In practical terms, this approach translates hydrogel chemistry into tunable network density, gelation kinetics, and matrix stiffness under cytocompatible conditions. A representative approach is strain-promoted azide-alkyne cycloaddition (SPAAC), in which strained cyclooctyne derivatives (e.g., DBCO or BCN) react with azide-functionalized polymers to form stable 1,2,3-triazole linkages without metal catalysts. The intrinsic ring strain lowers the activation energy, enabling rapid gelation under physiological conditions (37 °C, aqueous environment), which is particularly suitable for encapsulating sensitive human iPSC-derived neural progenitors.

Because SPAAC reactions proceed independently of endogenous cellular processes, crosslink density can be precisely tuned by controlling functional group stoichiometry. In brain organoid-oriented systems, this strategy could enable stage-specific modulation of matrix stiffness, maintaining a compliant microenvironment during early neuroepithelial expansion and potentially reinforcing the network during later endothelial lumen formation and blood–brain barrier maturation processes. Thus, click-mediated crosslinking provides a useful engineering framework for coupling hydrogel chemistry with matrix mechanics, offering significant implications for neurovascular development [47,48].

In parallel, 3D bioprinting offers an engineering-driven strategy to predefine vascular architecture [49]. Sacrificial scaffold approaches generate hollow microchannels within bulk hydrogels, which can be endothelialized to create perfusable conduits [50,51]. When combined with enzyme-responsive or reversible matrices, this hybrid system enables immediate perfusion while preserving long-term remodeling capacity in response to organoid growth and angiogenic signaling [52].

The engineering parameters and biological implications of these diverse platforms are synthesized in Table 2, providing a comparative overview of how different triggering mechanisms–including light, enzymatic activity, temperature shifts, reversible supramolecular interactions, and bio-orthogonal chemistry–modulate key matrix properties such as stiffness, degradability, and stress relaxation. By mapping these technical attributes to specific functional outcomes in brain organoid models, the table facilitates a direct comparison between various hydrogel strategies. This synthesis also provides a conceptual framework for the following section, which evaluates these platforms from a critical, brain organoid-specific perspective.

### 3.5. Comparative Perspective for Brain Organoid Vascularization

While photoresponsive hydrogels provide unmatched spatial precision for on-demand microchannel formation and stiffness modulation, they also enable localized control of matrix architecture. Such features are particularly advantageous for directing endothelial invasion into dense organoid cores. However, photoinitiator-associated cytotoxicity and limited light penetration remain important constraints when applied to large three-dimensional constructs.

In contrast, enzyme-responsive hydrogels rely on endogenous cellular activity, enabling self-regulated matrix remodeling that more closely resembles physiological angiogenesis. This cell-driven remodeling supports gradual vascular integration during long-term organoid maturation, although the kinetics of degradation and network formation are more difficult to control externally.

Thermo-responsive and supramolecular matrices primarily influence cell distribution, matrix viscoelasticity, and stress relaxation rather than directly defining vascular migration. However, these systems can improve nutrient diffusion and enable gentle cell encapsulation within soft microenvironments.

From a brain organoid perspective, these hydrogel platforms should not be evaluated solely on their responsiveness to external stimuli, but rather on their ability to support prolonged neural culture, endothelial specialization, astrocyte–pericyte integration, and the maintenance of low-stiffness yet mechanically stable microenvironments.

Taken together, these strategies present distinct advantages and limitations depending on the developmental stage of the organoid and the level of experimental control required. Photoresponsive systems provide precise spatial patterning but face penetration and cytotoxicity constraints; enzyme-responsive systems more closely approximate cell-driven angiogenic remodeling but offer less external control; and thermo-responsive or supramolecular matrices favor stress relaxation and gentle encapsulation, although they may provide less architectural precision or long-term structural robustness.

Therefore, the suitability of each hydrogel strategy ultimately depends on the developmental stage of the organoid, the desired level of spatial control, and the balance between experimental tunability and physiological relevance.

### 3.6. Experimental Evidence Supporting Dynamic Hydrogel-Mediated Vascularization

Recent experimental studies have begun to validate the practical potential of dynamic hydrogel systems for supporting vascular morphogenesis in 3D tissue models. For example, enzymatically degradable PEG-based matrices incorporating matrix metalloproteinase (MMP)-sensitive crosslinkers have demonstrated that tuning hydrogel degradability and cell-adhesive ligands enables robust endothelial invasion and the formation of perfusable microvascular networks in 3D culture systems [36,55,56].

Complementarily, visible light-responsive hydrogels incorporating photo-activated moieties now allow spatially confined modulation of matrix viscoelasticity. Such photocontrolled mechanics are particularly relevant to neural tissue engineering, where matrix viscoelasticity has been shown to regulate human neural stem cell differentiation and neural maturation, although direct validation in vascularized brain organoid systems remains limited [35,54].

Beyond synthetic scaffolds, matrix remodeling-dependent lumen formation has been reported in angiogenic sprouting models, emphasizing the necessity of dynamic degradation during network assembly. Notably, planar neural organoids assembled on PEG hydrogels have been reported to support endogenous CD31+ vascular-like networks whose expansion appears to depend on VEGF signaling.

Collectively, these findings support the practical relevance of dynamic hydrogel design principles, including enzymatic degradability and adaptive viscoelasticity, both of which promote vascular morphogenesis in related 3D culture systems and emerging neural organoid models [18,57].

These studies underscore the transition from theoretical biomaterial design toward experimentally informed neurovascular modeling strategies.

## 4. Advanced Strategies for Spatiotemporal Vascularization

### 4.1. Stress-Relaxing Hydrogels: Viscoelasticity-Driven Sprouting

Recent frontier studies emphasize that the viscoelasticity (stress relaxation) of the gel is as important as its elasticity. This property may be particularly relevant in brain organoid systems, where endothelial invasion must occur without mechanistically disrupting fragile neural progenitor zones and developing neuronal networks. In related vascularized hydrogels, fast stress relaxation rates reduce resistance to EC invasion and have been associated with purely elastic gels of the same stiffness [58,59].

### 4.2. Sequential Release of Pro-Angiogenic Factors

Because vascularization is a staged process, dynamic platforms are increasingly designed to regulate the temporal presentation of pro-angiogenic cues through controlled or sequential release systems. In principle, dynamic platforms can be designed so that the initial release of vascular endothelial growth factor (VEGF) promotes early endothelial sprouting and migration, followed by delayed delivery of Angiopoietin-1 (Ang-1) and Platelet-derived growth factor (PDGF) to support vessel maturation [60]. These factors are critical for recruiting pericytes and reinforcing nascent vessels, thereby preventing the structural instability and chaotic morphology typically observed in disordered sprouting.

### 4.3. In Situ Spathial Patterning: On-Demand Vascularization

One of the major barriers to scaling brain organoids is central hypoxia followed by necrotic core formation. To circumvent this, on-demand vascular path generation has emerged as a transformative strategy [21]. Unlike static scaffolds, this approach allows researchers to intervene at critical developmental stages: once the organoid reaches a specific volume, localized laser irradiation is employed to induce site-specific softening of the hydrogel [61].

This photopatterning technique can facilitate the creation of oxygen diffusion conduits and artificial micro-channels, potentially providing low-resistance paths for endothelial cell (EC) infiltration [62]. By generating these oxygen supply routes when and where metabolic demand increases, this method may offer substantial spatiotemporal control and help improve the long-term viability and physiological relevance of large-scale 3D brain models [63].

Despite their potential, these advanced vascularization strategies also present several limitations. Photopatterning approaches may introduce phototoxicity and limited light penetration in larger constructs, while sequential growth factor delivery requires precise temporal control to avoid aberrant angiogenesis. In addition, microfabrication or bioprinting-based vascular architectures often increase experimental complexity and may reduce reproducibility across laboratories.

## 5. Biological Impact on Neurovascular Unit (NVU) Maturation: Functional Synergy

A major goal of advanced brain organoid engineering is to move beyond simple neural aggregates toward more functionally integrated models that better approximate the structural and cellular complexity of native brain tissue through progressive NVU assembly [64]. The NVU represents a fundamental functional unit comprising brain microvascular endothelial cells, pericytes, astrocytes, and neurons, all anchored by a supporting basement membrane. Beyond its role in establishing the blood–brain barrier (BBB), the NVU is pivotal in regulating neural metabolism and maintaining brain homeostasis through complex paracrine signaling [65].

In the in vivo microenvironment, NVU maturation is a spatiotemporally regulated process driven by dynamic mechanical stimuli, precise cell–matrix interactions, and angiocrine signaling cascades [26,66,67]. Conventional static hydrogels, such as Matrigel or fixed synthetic scaffolds, are fundamentally limited in capturing these developmental dynamics due to their invariant physical properties [26]. In contrast, stimuli-responsive biomaterial platforms offer a paradigm shift; by enabling on-demand control over matrix stiffness and the sequential release of biochemical cues, they precisely mimic the evolving environment of the maturing human brain [68]. Consequently, these dynamic platforms should be viewed as promising next-generation modeling tools that may support aspects of NVU maturation, extending investigation beyond simple vascularization toward more biomimetic brain models.

### 5.1. BBB Permeability and Tight Junctional Reinforcement

As the NV matures, mechanotransduction pathways such as YAP/TAZ have been shown to respond to matrix stiffening in endothelial culture systems, and may play a role in BBB maturation within organoid models [69,70,71,72,73]. In related endothelial culture systems, such mechanical instruction has been associated with upregulation of key tight junction proteins, including ZO-1, Occludin, and Claudin-5, and may contribute to barrier reinforcement (high trans-endothelial electrical resistance, TEER) and selective permeability in vascularized organoid models [26].

### 5.2. Neurovascular Synergism: Angiocrine Signaling

The establishment of vascular-like networks within dynamic gels may create a localized signaling interface between endothelial and neural compartments. In related neurovascular and organoid systems, endothelial cells have been reported to secrete angiocrine factors (e.g., BDNF, IGF, TGF-β) that may promote neural progenitor proliferation and synaptic maturation [64,74]. By better coordinating angiogenic and neurogenic processes, dynamic hydrogels may promote neurovascular synergism, reduce central metabolic stress, and improve aspects of organoid maturation [63,75].

### 5.3. Dynamic Disease Modeling: Degeneration and Regeneration

The inherent reversibility of stimuli-responsive hydrogels offers a potentially useful platform for temporally controlled modeling of neurovascular pathologies. By triggering on-demand matrix degradation or stiffness loss, researchers can simulate the acute BBB breakdown observed in stroke or Alzheimer’s disease [76]. Subsequent therapeutic intervention or matrix re-stabilization then provides a unique window to observe vascular and neural regeneration, offering a robust platform for personalized medicine and drug screening [77].

## 6. Structural and Functional Characterization

**Figure 2:**
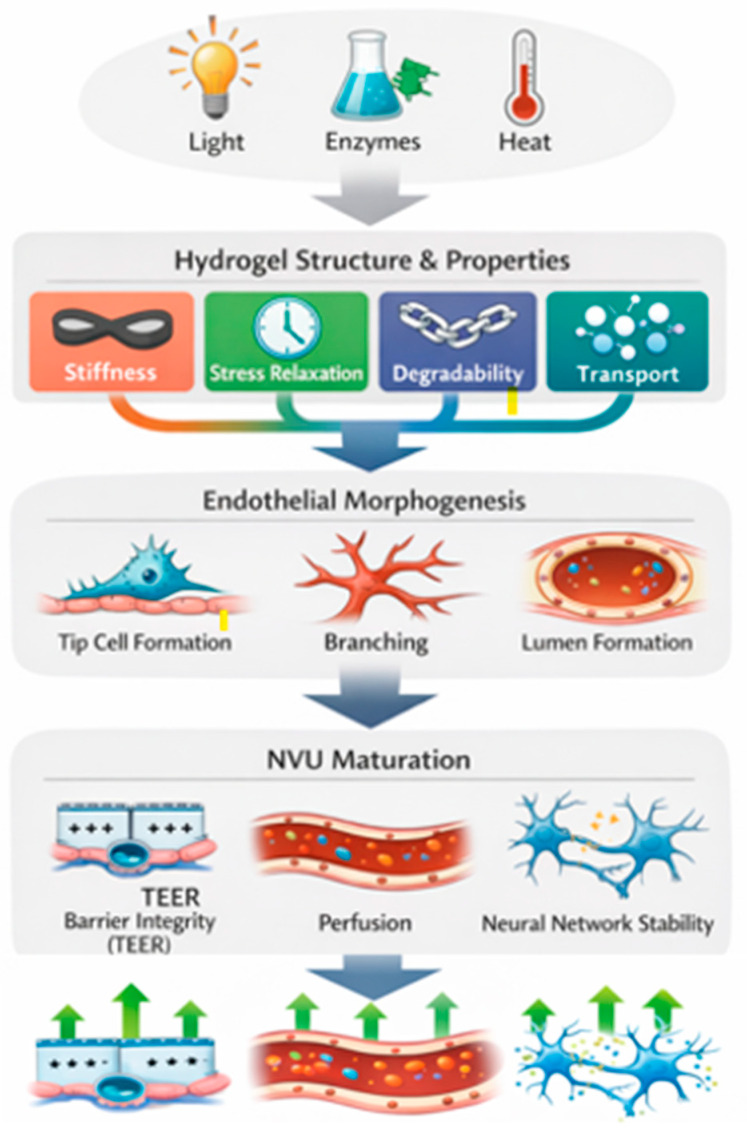
The structure–property–function framework underlying dynamic hydrogel-mediated in neurovascular assembly. TEER: trans-epithelial electrical resistance. The arrow indicates increase and activation.

The evaluation of brain organoids cultured in dynamic hydrogels must extend beyond morphological description to determine how the engineered microenvironment directs tissue maturation. These responsive biomaterial systems are best understood through a structure–property–function axis in which hydrogel chemistry and the crosslinking strategy determine matrix stiffness, stress relaxation, degradability, and transport behavior, which regulate endothelial morphogenesis, including tip-cell formation, branching behavior, and lumen development, and ultimately shape NVU maturation, influencing barrier integrity, perfusion capacity, and neural network stability. As schematized in Figure 2, the evaluation of brain organoids within these environments must move beyond simple morphological observations to quantify how this engineered axis actively dictates tissue maturity.

Stimuli-responsive cues (e.g., light, enzymatic activity, or thermal triggers) modulate key matrix properties, including stiffness, stress relaxation, and degradability. These biomechanical changes direct endothelial morphogenesis such as tip cell formation, branching, and lumen development to ultimately promote functional NVU maturation, characterized by enhanced barrier integrity (TEER), perfusion, and synaptic integration.

**Figure 3:**
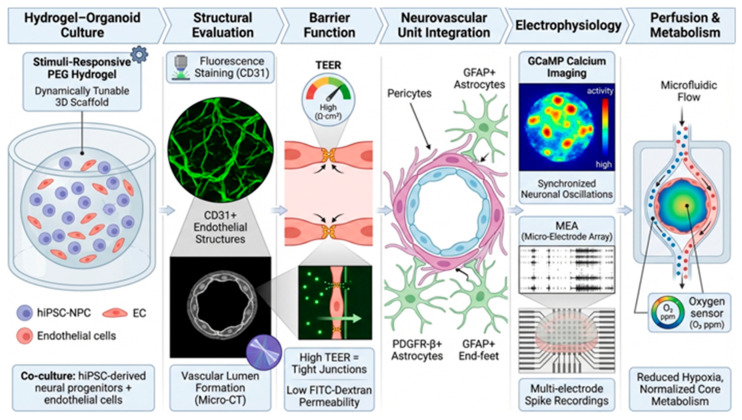
Graphical summary of the evaluation workflow for vascularized brain organoids cultured in dynamic hydrogel systems. Arrows indicate the sequential workflow and functional relationships. Colors denote specific cell types and structures: neural progenitors (blue/purple), endothelial cells (red), astrocytes (green), and neurovascular unit components, with tight junctions shown in yellow and permeability assessed using fluorescent tracers. hiPSC-NPC: human induced pluripotent stem cell–derived neural progenitor cells, EC: endothelial cells, PDGFR-β: platelet-derived growth factor receptor beta, GFAP: glial fibrillary acidic protein.

To provide an integrated overview of these evaluation strategies, a graphical summary of the characterization workflow is presented in Figure 3.

Structural and functional characterization proceeds through multiple complementary levels of analysis. Vascular morphology is first evaluated through fluorescence staining of endothelial markers such as CD31 and visualization of lumen formation. Barrier integrity is subsequently quantified using TEER measurements and permeability assays. Neurovascular unit (NVU) integration is assessed through pericyte coverage and astrocytic end-foot association. Electrophysiological activity is analyzed using calcium imaging and multi-electrode array (MEA) recordings. Finally, perfusion and metabolic support are evaluated using microfluidic flow systems and oxygen sensing to assess tissue viability and metabolic stability.

### 6.1. Imaging and Vascular Metrics

The structural integration of vascular networks within dynamic hydrogels is evaluated using high-resolution three-dimensional imaging, typically confocal or multiphoton microscopy, to visualize CD31 (PECAM-1)- and Lectin-positive endothelial structures. Lumen formation, network continuity, and spatial organization are assessed alongside basement membrane deposition, including Laminin and Collagen IV [78,79,80,81].

In dynamic matrices, vascular networks are characterized by their ability to extend toward and penetrate the organoid core. This progression is quantified using total vessel length, mean vessel diameter, and branching point density, enabling direct comparison with static controls [57,82].

Mechanical modulation contributes to these structural outcomes. In related hydrogel-based vascularization studies, stiffness-tuning experiments have shown that localized photo-softening from 10 kPa to 1 kPa during defined developmental stages increases vascular branching density relative to static high-stiffness conditions [14]. These observations support the concept of biphasic mechanical requirements in which relatively higher early matrix supports may support tissue organization, whereas softening may facilitate endothelial tip-cell invasion and network expansion [23,83].

### 6.2. Barrier Integrity and Selective Permeability

Functional maturation of vascularized organoids requires establishment of a selective and electrically stable endothelial barrier. Transendothelial electrical resistance (TEER) provides a quantitative measure of ionic sealing between adjacent endothelial cells and serves as a primary indicator of barrier integrity [84,85]. TEER values should be interpreted relative to physiological benchmarks. In vivo human BBB resistance is generally estimated to exceed 1500–2000 Ω·cm^2^ [86], whereas most organoid-based in vitro systems report lower values. Comparison with established human BBB models therefore provides important context for evaluating the extent to which dynamic hydrogel platforms approximate physiological barrier tightness. Increased TEER values in dynamic hydrogel systems are associated with continuous localization of tight junction proteins, including ZO-1 and Claudin-5 [85,87,88].

Barrier selectivity is further evaluated using permeability assays with fluorescent dextrans of defined molecular weights or comparable tracer compounds [89]. Restricted diffusion from the intraluminal compartment into the surrounding tissue indicates reduced paracellular transport [89,90]. Assessment of BBB-associated transporters such as GLUT-1 and P-gp provides additional molecular evidence of barrier specialization [91,92].

### 6.3. NVU Cellular Integration and Niche Assembly

A functional neurovascular unit (NVU) depends on coordinated recruitment of supporting cells to the endothelial basement membrane. Characterization includes quantification of pericyte coverage along vascular walls and evaluation of polarized astrocyte end-foot contact with the endothelial lumen, both of which contribute to vascular stabilization and barrier refinement [65,93].

Within dynamic hydrogel systems, this multicellular organization is accompanied by the induction of BBB-associated transporters, including GLUT-1 and P-gp [91,92]. Such expression patterns are consistent with the establishment of a specialized neurovascular niche rather than a structurally isolated endothelial network.

### 6.4. Electrophysiology and Network Synchronization

Electrophysiological profiling provides a functional readout of vascular integration. Calcium imaging and multi-electrode array (MEA) recordings are used to assess spontaneous firing activity, burst frequency, and network synchronization within organoids [94,95,96].

Comparative studies report differences in firing stability and synchronized burst dynamics between vascularized and non-vascularized constructs [74,97]. These observations are discussed in relation to improved metabolic support and endothelial–neural signaling at the neurovascular interface.

Matrix mechanics may further influence neuronal behavior. Modulation of hydrogel viscoelasticity alters stress relaxation dynamics and in related mechanobiology studies, has been proposed to influence endothelial secretion of angiocrine factors, including BDNF and GDNF [23,98]. These signaling pathways have been associated with neural stem cell proliferation and differentiation in viscoelastic environments approximating native brain tissue [74,97,99].

### 6.5. Perfusion, Metabolic Efficiency, and Viability

While endothelial marker expression and lumen-like morphology indicate structural vascularization, these features alone do not confirm functional perfusion. True vascular functionality requires evidence of continuous intraluminal flow, shear stress responsiveness, and tracer circulation under dynamic conditions.

Functional validation requires evidence of sustained perfusion and metabolic stability within the engineered construct. Integration with microfluidic platforms enables application of biomimetic shear stress and continuous intraluminal flow [10,19]. Structural integrity of engineered vessels under perfusion is evaluated to assess mechanical robustness [14,19].

Perfusion efficiency is examined through tracer circulation assays and analysis of hypoxia-associated markers [100,101]. Enhanced oxygen and nutrient distribution are associated with reduced central necrosis and improved long-term tissue viability, addressing a recognized limitation of static organoid cultures [82].

Long-term validation represents an additional benchmark of functional relevance. Although vascular patterning is often reported within weeks, fewer studies demonstrate sustained perfusion and barrier stability beyond three months of culture [97,102]. An extended longitudinal assessment is required to confirm the durability of engineered neurovascular systems.

## 7. Challenges, Limitations, and Future Directions

Despite their promise in neurovascular unit (NVU) modeling, dynamic hydrogels still face translational limitations.

### 7.1. Current Challenges

The reliance on externally applied triggers, such as patterned light or chemical inputs, adds operational complexity that may constrain scalability and inter-laboratory reproducibility. In photoresponsive systems, synthetic photoinitiators including Irgacure derivatives and lithium phenyl-2,4,6-trimethylbenzoylphosphinate (LAP) are widely used to achieve spatiotemporal control of crosslinking; however, radical-mediated polymerization may generate reactive oxygen species (ROS) during irradiation [103,104]. Such oxidative stress can compromise the viability of sensitive human iPSC-derived endothelial and neural populations and may adversely affect mitochondrial function, barrier integrity, and long-term culture stability [105]. Furthermore, incomplete radical quenching or residual initiator fragments raise concerns regarding cumulative cytotoxicity in prolonged 3D culture systems.

Although copper-free click chemistries such as strain-promoted azide–alkyne cycloaddition (SPAAC) are generally regarded as more biocompatible, residual reactive moieties, side products, or multi-step functionalization strategies may still influence matrix stability and cellular responses over extended culture periods. From a translational perspective, additional challenges arise with respect to Good Manufacturing Practice (GMP) compliance, including batch-to-batch variability, sterilization compatibility, and regulatory classification of multifunctional biomaterials [27,106]. Careful evaluation of cytotoxicity, oxidative stress burden, and manufacturing robustness will therefore be essential before dynamic hydrogel platforms can be widely adopted for clinical-grade vascularized organoid applications.

### 7.2. Limitations

Several intrinsic limitations of dynamic hydrogel systems should also be acknowledged.

First, increased structural complexity and the risk of over-engineering stem from multi-component crosslinking and stimulus-responsive designs. While these approaches improve experimental control, they can reduce robustness and make outcomes sensitive to small procedural variations. Second, inter-laboratory reproducibility remains a concern, as minor differences in stimulus intensity, polymer modification, or reaction conditions may lead to inconsistent remodeling behavior and limit cross-study comparison. Finally, economic and translational feasibility relates to the cost of custom peptide synthesis, specialized photoinitiators, and multi-step fabrication, as well as the difficulty of translating highly customized materials into standardized, GMP-compatible products for industrial production. Without greater emphasis on simplification and standardization, dynamic hydrogels may remain valuable research tools but face challenges in broader clinical translation.

### 7.3. Future Directions

Next-generation platforms will likely shift toward autonomous, cell-responsive “smart” hydrogels that remodel in situ through endogenous cues such as protease activity or metabolic signals, reducing reliance on external triggers. Such biologically gated materials could create self-regulating microenvironments and reduce the need for external intervention.

Ultimately, integrating patient-derived iPSCs with dynamically tunable yet scalable scaffolds will advance high-fidelity vascularized brain models for precision disease modeling, drug screening, and regenerative medicine.

## 8. Conclusions

This review highlights dynamic hydrogels as a central enabling technology for advancing brain organoid maturation, particularly for functional neurovascular unit (NVU) reconstruction.

### 8.1. From Static Scaffolds to Evolutionary Microenvironments

Dynamic hydrogel platforms mark a decisive transition from conventional static scaffolds toward evolutionary microenvironments that adapt in concert with the temporal progression of brain organoid development. By overcoming diffusion constraints and fixed mechanical limitations inherent to traditional matrices, these systems better recapitulate the dynamic remodeling processes required for coordinated neurovascular maturation.

### 8.2. Integration of Stimuli-Responsive Mechanisms and Functional Maturation

The incorporation of stimuli-responsive mechanisms enables precise biomechanical and biochemical regulation of the organoid microenvironment. Controlled stiffness modulation may promote endothelial branching and vascular network complexity, while sequential growth factor release may support temporally defined angiogenic progression and blood–brain barrier stabilization, potentially improving barrier integrity and increasing TEER-related functional readouts.

### 8.3. Impact on NVU Crosstalk and Translational Relevance

By providing a permissive and programmable extracellular microenvironment, dynamic hydrogels enhance bidirectional neurovascular crosstalk and accelerate neural circuit maturation. The ability to reversibly tune matrix properties further enables modeling of vascular degeneration and regeneration processes associated with neurodegenerative diseases and post-stroke recovery, strengthening the translational relevance of vascularized brain organoids.

### 8.4. Future Horizons: Autonomous and Clinical Platforms

Looking forward, the integration of closed-loop regulatory strategies with cell-autonomous, biologically gated hydrogel systems may enable self-adjusting platforms that respond dynamically to organoid maturation states. Such developments could further enhance the stability, reproducibility, and physiological relevance of vascularized, patient-derived brain organoids, strengthening their utility as pre-clinical models for drug screening and regenerative research.

## Figures and Tables

**Table 1:** Comparison between conventional static matrices and emerging dynamic platforms for brain organoid vascularization.

Comparison Category	Static Hydrogels	Dynamic Hydrogels (Stimuli-Responsive)
Mechanical properties	Fixed stiffness; invariant over time	Tunable/on-demand stiffening or softening
Matrix remodeling	Passive; slow bulk degradation	Active; cell-instructed/reversible crosslinking
Vascular sprouting	Limited by physical/stiffness barriers	Accelerated via local softening or stress relaxation.
Nutrient & oxygen supply	Diffusion-limited (necrotic core > 200 μm).	Enhanced via perfusable or sacrificial channels
Biochemical signaling	Constant ligand presentation	Sequential/programmed growth factors (VEGF, Ang-1)
Biological relevance	Low mimicry of developmental dynamics	High mimicry of NVU maturation processes
Key challenges	Batch variability; diffusion bottlenecks	Complex orchestration; potential chemical toxicity

VEGF: Vascular Endothelial Growth Factor, Ang-1: Angiopoietin-1, NVU: neurovascular unit, μm: micrometer.

**Table 2:** Engineering strategies for dynamic and stimuli-responsive hydrogels in NVU maturation.

Strategy	Representative Hydrogel Systems	Trigger/Mechanism	Matrix-Level Modulation	Functional Outcome in NVU/Brain Organoids	Representative Models
Photoresponsive	PEG-NB, MeHA	UV/visible light (laser patterning)	Local stiffness (ΔE) modulation; photo-degradable patterning	Guided vessel infiltration; spatial oxygen control	Brain organoid vascular infiltration models [18,53,54]
Enzyme-responsive	MMP-cleavable PEG, GelMA (photo-crosslinkable and MMP-degradable)	Cell-secreted proteases (MMPs)	Enzymatic degradability; pore expansion	Synchronized NVU coupling; cell-instructed remodeling	Angiogenic sprouting models [34,35,36]
Thermo-responsive	pNIPAM	Temperature shift	Reversible sol–gel transition; diffusivity control	Homogeneous 3D cell distribution; nutrient transport	Human iPSC-derived neural models [39,40]
Supramolecular	β-CD/adamantane, Alginate	Non-covalent reversible bonding	Viscoelasticity; stress relaxation	Stress-adaptive matrix for EC sprouting; self-healing	3D vascular network assembly [42,43]
Bio-orthogonal/Click	SPAAC PEG, tetrazine ligation systems	Chemical cues (click chemistry)	Crosslink density control; biofunctionalization	In situ mechanical tuning; growth factor release	Neuroepithelial expansion & maturation [44,45]
3D Bioprinted	Sacrificial pluronic templates	Thermal/chemical removal	Predefined microarchitecture; hollow micro-channels	Predictable vascular architecture; perfusable conduits	Perfusable vascularized brain organoid models [47,48,49]

ΔE denotes changes in Young’s modulus; PEG-NB: Polyethylene Glycol Norbornene; MeHA: Methacrylated Hyaluronic Acid; EC: endothelial cell; NVU: neurovascular unit; MMP: Matrix Metalloproteinase.

## Data Availability

No new data were created or analyzed in this study. Data sharing is not applicable to this article.
